# Lectin-mediated reversible immobilization of human cells into a glycosylated macroporous protein hydrogel as a cell culture matrix

**DOI:** 10.1038/s41598-017-06240-w

**Published:** 2017-07-21

**Authors:** Nicholas Bodenberger, Dennis Kubiczek, Laura Trösch, Ali Gawanbacht, Susanne Wilhelm, Denis Tielker, Frank Rosenau

**Affiliations:** 1 Center for Peptide Pharmaceuticals, Faculty of Natural Science, Ulm University, Ulm, 89081 Germany; 2 Core Facility Flow Cytometry, Ulm University, Ulm, 89081 Germany; 3 Heinrich Heine Universität Düsseldorf, Deans Office, Düsseldorf, 40204 Germany; 4 QIAGEN GmbH - Germany, Qiagen Straße 1, Hilden, 40724 Germany

## Abstract

3D cell culture is a helpful approach to study cell-cell interaction in a native-like environment, but is often limited due the challenge of retrieving cells from the material. In this study, we present the use of recombinant lectin B, a sugar-binding protein with four binding cavities, to enable reversible cell integration into a macroporous protein hydrogel matrix. By functionalizing hydrogel precursors with saccharose, lectin B can both bind to sugar moieties on the cellular surface as well as to the modified hydrogel network. Confocal microscopy and flow cytometry analysis revealed cells to be integrated into the network and to adhere and proliferate. Furthermore, the specificity and reversibility was investigated by using a recombinantly produced yellow fluorescent - lectin B fusion protein and a variety of sugars with diverging affinities for lectin B at different concentrations and elution times. Cells could be eluted within minutes by addition of L-fucose to the cell-loaded hydrogels to make cells available for further analysis.

## Introduction

Modern biotechnology is a field which cannot be imagined without cell culture – as a model for diseases, tumors, as a production system for cells or recombinant products, or for initial drug testing^1–3^. Although 2D cell culture systems have been state of the art for many years, they are often limited by certain factors; cells can only grow to a given spatial density and upon formation of a monolayer cells will detach or die. Furthermore, cell contacts are limited to those possible in the artificial 2D environment, diffusion of compounds and growth factors is vastly different from natural environments and the growth substratum consists of synthetic materials (e.g. plastic dishes, flasks). Cells with special demands for physiologic niches may not be provided with the required microenvironment concerning e. g. mechanical properties^4–6^. One approach to overcome these considerable limitations is the development of 3D cell culture matrices to provide optimized growth conditions and to mimic physiologic cell environments. Many different systems have been developed in recent years, each with its own advantages and limitations.

Direct cell encapsulation during hydrogel formation leads to an easy handling and a uniform distribution of cells within the materials, but is often challenging due to cell toxicity effects of precursors and the inherent lack of reversibility. Disintegration of the polymer networks within hydrogels, as it may be required for the release of cells for certain applications, can be triggered by different methods using temperature changes^7, 8^, reversible protein or peptide interaction^9^, enzymatic degradation^10^, changing pH^11^, addition of denaturing agents^11^ or by electrochemical stimulation^12^. Thus, the key challenge is to produce systems from biocompatible precursors, and both gel formation and disintegration for cell release purposes have to be fast, gentle and cell compatible.

Another approach to fabricate matrices for 3D cell culture is the formation of macroporous systems which allow subsequent infiltration of cells after material formation, thereby separating matrix formation and seeding of the cells. Elaborate 3D architectures are formed by methods like particle-leaching^13^, freeze-drying^14^ or advanced techniques like lithography and spin-coating as nicely reviewed by Selimović *et al*. in ref. 15. Those materials are often stable and cell-compatible as cells are added after polymerization of hydrogels and any possibly harmful components can be removed prior addition of cells.

Common precursors for hydrogel formation are natural precursors like proteins (collagen, elastin and elastin-like peptides, fibrin and lysozyme)^16–22^, DNA^23^, polysaccharides (dextran, alginate, hyaluronic acid)^24–26^, artificial components (polyethylene glycol, methacrylamide^10, 27–30^) or a mixture of different types of chemical or biological components. In this study, we aimed to introduce a new method for the reversible immobilization and adhesion of cells into a macroporous hydrogel system based on reversible binding of cells to sugar residues mediated by a multivalent lectin as a specific molecular adapter.

Lectins are sugar-binding proteins which are ubiquitously distributed in all kingdoms of life and can specifically bind to glycosylated structures on or within cells, have no enzymatic activity but are involved in several cellular processes (e. g. directed trafficking of proteins)^31, 32^. Here, we have used a lectin variant consisting of the bacterial lectin B (PA-IIL) domain^33^ fused to a yellow fluorescent protein (YFP) reporter domain as a fluorescent reporter which forms a homotetramer harbouring four sugar-binding sites composed of the c-terminal parts of two subunits each^33^. Native LecB has the highest affinity for L-fucose (KA = 1.6 × 10^6^/M) and decreasing, but still high affinities for L-galactose, D-mannose and D-fructose^33^. Tielker *et al*. have recombinantly produced LecB in *Escherichia coli* and established a purification method through mannose agarose beads to explore the potential of LecB as a tag for single-step protein purification^34^. Furthermore, they constructed a YFP-LecB fusion protein containing LecB as a binding domain and YFP as a reporter for different possible applications. The different sugar affinities of this fusion protein can now be used to reversibly immobilize cells into a 3D matrix.

As a 3D scaffold and substratum for the lectin mediated immobilization of human cells, a protein based macroporous hydrogel system was used which we have recently developed. It has been shown to provide adjustable pore sizes and tunable mechanical properties for cell culture experiments^35^. This material is composed of human serum albumin, which is covalently crosslinked via addition of tetrakis (hydroxymethyl) phosphonium chloride (THPC) as a four armed linker. This results in pore sizes in the low nanometer range^32^. Larger – and thus more suited for resembling niches in cell culture - pores in the mid micrometer range can easily be prepared from this by leaching techniques or freeze-drying and subsequent reswelling^36^. This material was modified by introducing sugar residues to the BSA as the protein ingredient prior to crosslinking using a very simple method for the glycosylation of proteins under dry conditions^37, 38^. This initial step introduced freely accessible fructose adapter moieties from saccharose molecules linked via the glucose residue to the protein backbone to enable lectin binding and decoration of the material. Lectin bound to the protein backbone in this way was quantitatively eluted by the addition of fucose which is a ligand of YFP-LecB with higher affinity than fructose. The tetrameric structure of the active YFP-LecB resulted in simultaneous binding to cells and to the glycosylated hydrogel scaffold, thereby acting as a molecular cell immobilizer. Lectin based cell immobilization was reversible by elution with fucose and the cells incorporated in the material via YFP-LecB, were able to proliferate in the matrix prior to elution as was shown by confocal microscopy and by flow cytometric analysis of the eluted cells. Harvested cells kept their ability to proliferate and showed an unaltered over all morphology compared to conventionally grown controls. Although this technique is based on lectin-sugar interactions which definitively differ from natural interactions between cells and matrix components, we believe that this novel system may be helpful to develop processes for the production of cells in which their initial attachment to a 3D matrix is required. Special bioreactors for the growth of cells can be imagined that may allow iterative rounds of proliferation and harvesting of cells with requirements for special niches resembling their mechanical properties, which may be reconstructed in and thus provided by the 3D matrix presented here in combination with the gentle recovery by sugar elution.

## Results

Lectin B is a four-armed sugar binding protein with different affinities for a variety of sugars, with moderate affinities for glucose and fructose and high affinity for fucose. In order to realize binding of YFP-LecB to the macroporous cell culture matrix composed of chemically crosslinked BSA, we used the sugar dimer saccharose to introduce a receptor. The carbonyl group of the glucose moiety of saccharose can bind to the backbone of the hydrogel material in a Maillard-like reaction^38, 39^. This results in an accessible fructose residue, which is now available for subsequent decoration with lectin. The use of saccharose leads to a major advantage: lectin can bind to the fructose with moderate affinity. Many sugars with higher affinity for lectin B are available, like mannose or fucose which can competitively bind to the lectin to elute the protein from the matrix.

**Figure 1:**
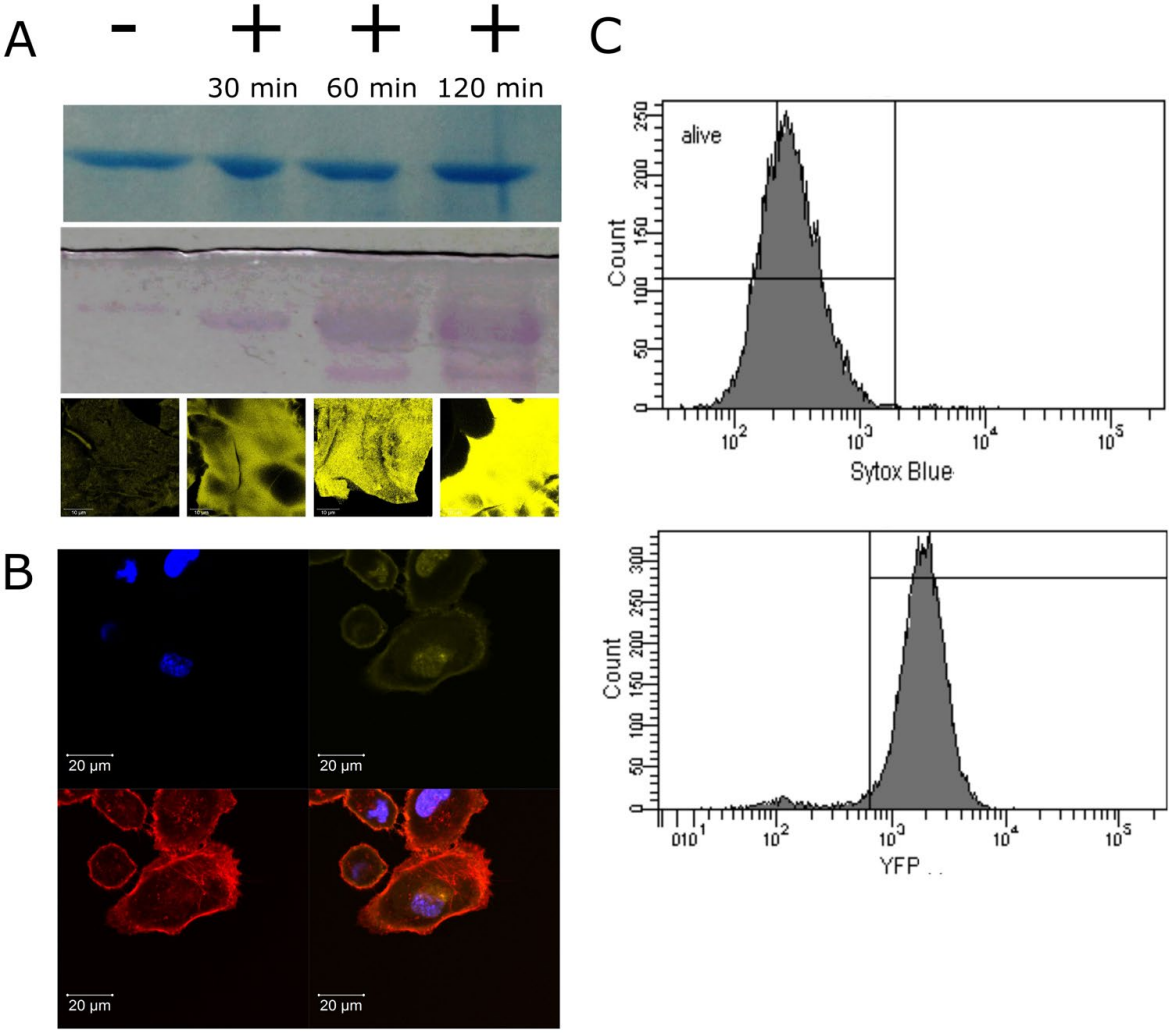
Decoration of cells and hydrogels with YFP-LecB and biocompatibility. (**A**) The glycosylation of BSA was determined with an acid-schiff reaction. BSA was analysed on a SDS page (**A** top) and a glycoprotein detection gel (middle) for different glycosylation times (30, 60 and 120 min) where glycosylation is visualized by magenta bands in the gel (shown are only the relevant parts from full length gels in Fig. S4 Supplementary Information). Confocal images (lower panel) of YFP-LecB decorated hydrogels for untreated and glycosylated (30, 60 and 120 min) hydrogels which were incubated with YFP-LecB, followed by subsequent washing. Yellow color represents the bound YFP-LecB. (**B**) Lung cancer cells (A549) were incubated with YFP-LecB, washed and visualized with confocal microscopy to reveal lectin binding to the cells whereas red color represents the rhodamine-phalloidin stained cytoskeleton, blue the DAPI stained cell nucleus and yellow the YFP-LecB. (**C**) Cells were incubated with YFP-LecB and SytoxBlue to reveal the extent of YFP-LecB binding to the cells and its biocompatibility; YFP-LecB was proved to be biocompatible (overall viability > 95% after 24 h) and to bind to nearly all cells (bottom right).

Saccharose consists of glucose and fructose monomers; the carbonyl group of a sugar interacts with the nucleophilic amino groups of amino acids in a Maillard-like reaction^38^, binding a glucose molecule to the serum albumin and leaving a residual fructose moiety, which can be bound by lectin^38^. Glycosylation of bovine serum albumin (BSA) was shown using a periodic acid-schiff reagent method, based on the oxidation of hydroxyl groups of the sugar molecules and detection of the resulting aldehyde groups by the formation of purple colored bands in a sodium dodecyl sulfate (SDS) gel. Compared to a non-glycosylated control the intensity of this staining increased with the duration of glycosylation (Fig. 1A, lower panel). When the protein samples where stained with a Coomassie Blue stain as a simple and conventional protein staining method^40^ bands at the same height and thus the same molecular weight can be seen (Fig. 1A, top panel). On the other hand, there are significant differences in the intensity for the glycosylated proteins. For untreated BSA, only marginal staining was observed while glycosylation increased intensity with glycosylation time, reaching highest intensities after 120 minutes (Fig. 1A, middle panel). Both types of BSA – glycosylated and untreated – readily formed hydrogels when mixed with THPC as described in materials and methods within minutes and no differences in their appearance were observed. Both types of gels were analysed for their potential to bind YFP-LecB by confocal laser scanning microscopy (CLSM) which revealed a low signal for the untreated BSA hydrogel, which might be a result of residual trace amounts of glycoproteins in the BSA. According to the manufacturer, BSA is at least 98% pure, but as it is extracted from serum, there may be minor contaminations by glycoproteins including antibodies^41^, which can potentially be bound by sugar-binding lectins and thus may be the reason for minimal staining (Fig. 1A, lower panel). Compared to the BSA which was further glycosylated with saccharose, a clear difference can be seen as much more YFP-LecB can be detected on the surface of this hydrogel species with increased LecB binding for prolonged glycosylation times.

Furthermore, the potential of LecB to bind to surfaces of human cells was investigated. CLSM revealed a specific and durable interaction between lectin B and the cell surface of adenocarcinomic human alveolar basal epithelial cells (A549) (Fig. 1B) and human breast adenocarcinomic cells (MCF7) (Supplementary Fig. S1) It is well-characterized that cells contain a vast amount of different sugars – including glucose, galactose, mannose and fucose^42^ – as compounds of surface glycan which can be receptors for a large variety of lectin species^36, 43^. In this study we prove that our *Pseudomonas aeruginosa* derived YFP-LecB is capable of binding A549 and MCF7 cell lines with a high affinity within minutes. Native LecB had already been shown to bind to cell surface of e.g. lymphocytes^44^. Flow cytometry analysis revealed that basically all cells were decorated with lectin (Fig. 1C, lower panel and Supplementary Fig. S1), (94.9%). Furthermore, endotoxin levels were analysed and found to be 1.1 EU/ml in protein fractions, a concentration with no effect on human cells^45^. The lectin fusion protein itself proved to be biocompatible as no cytotoxicity was observed in a cell flow cytometry based assay, qualifying this protein a good candidate for a novel and non-toxic tool in cell culture studies.

**Figure 2:**
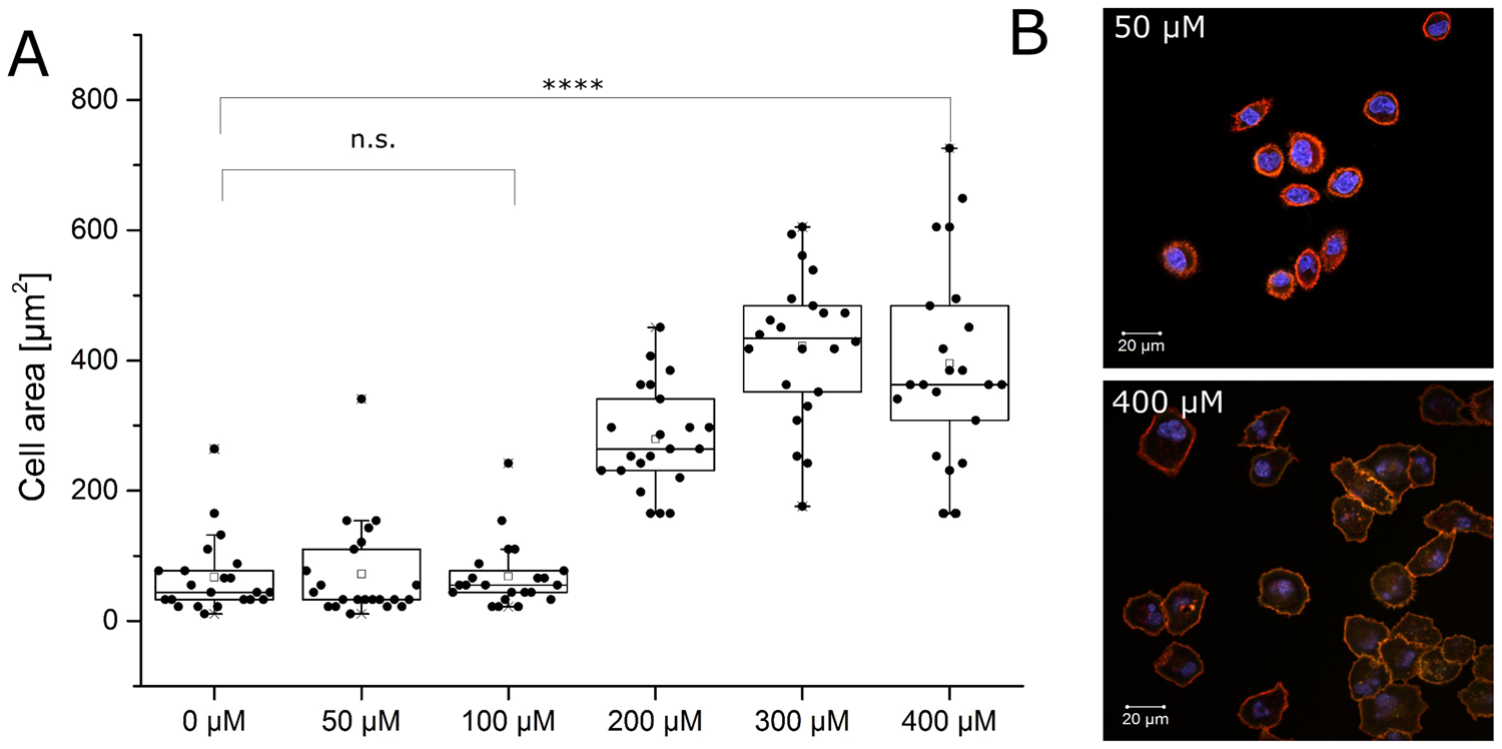
Lectin-mediated cell adhesion. (**A**) 2*10^5^ cells were seeded onto a hydrogel surface in the absence or presence of different YFP-LecB concentrations from 0 to 400 µM. Cells could adhere to the surface in the presence of cell culture medium for 24 h, followed by staining of cell cytoskeleton (red phalloidin-rhodamine) and nucleus (blue DAPI). Cellular surfaces were analysed with confocal laser scanning microscopy. Adhesion was detected for YFP-LecB concentration of 300 µM and higher. (**B**) Typical A549 cells in the presence of 50 and 400 µM of YFP-LecB. All bars represent the standard deviation. The significance was tested with a one way anova with alpha = 0.05.

Next, the potential of the lectins to mediate cellular adhesion to the hydrogel matrix was investigated. To do so, cells were seeded onto the hydrogel matrix in the presence and absence of YFP-LecB, with concentrations ranging from 0 to 400 µM. Cells were analysed after 24 hours of cellular growth on the hydrogel matrix by CLSM and significant differences in contact intensity were obvious. As a measure for this, the size of the cells was investigated with graphic analysis software (GSA Image Analyzer, GSA, version 419 3.8.7) and revealed efficient adhesion of the cells onto the hydrogel. Whereas cells in the absence of YFP-LecB had a surface of less than 100 µm^2^ in average, the YFP-LecB exposed cells showed surface areas increasing with the concentration of YFP-LecB up to more than 400 µm^2^ at concentrations higher than 300 µM (Fig. 2 and Supplementary Fig. S2). Although, the analysis of the adhesion of A549 cells is difficult since it is known to differ strongly on different types of surfaces^35^ the adhesion promoting influence of YFP-LecB is obvious. Furthermore, cells seeded onto the BSA hydrogel matrix as a control without lectin, easily detached from the surface by intense washing with PBS, which was not possible in the presence of YFP-LecB at concentrations higher than 100 µM.

**Figure 3:**
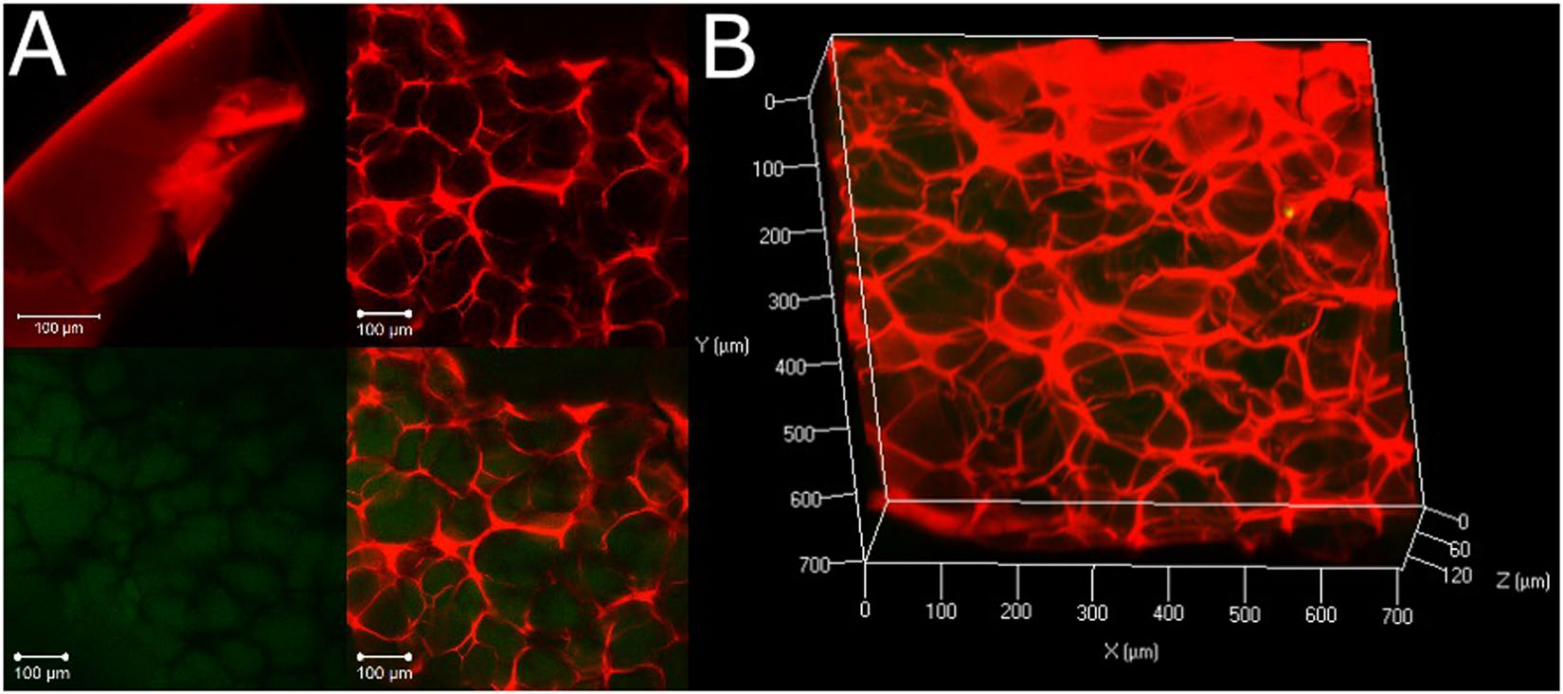
Hydrogel structure. Hydrogels were prepared by mixing both glycosylated protein backbone and linker, followed by polymerization. Hydrogels were incubated for 10 min with 300 µM YFP-LecB (red color) to show the protein binding to the glycosylated matrix. Hydrogels were visualized with confocal laser scanning microscopy. (**A**, Top left) Furthermore, hydrogels were freeze-dried to generate pores by evaporation of ice crystals from the matrix and analysed with confocal imaging (**A**, top right). To test interconnectivity of pores and specificity of YFP-LecB binding, hydrogels were diffused by a green fluorescent protein which is structurally similar to the yellow fluorescent protein. 200 µM of GFP could freely diffuse throughout the whole network and was only localized in the pores (**A**, bottom left and right). (**B**) A 3D image of a macroporous hydrogels in the presence of GFP.

Freeze-drying of hydrogels after polymerization was used to create simple 3D architectures^46, 47^. Ice crystal nucleation within the water-loaded environment of the material disrupts and replaces the initial hydrogel structure and leaves a porous network after sublimation of the ice from the material. Figure 3A shows the native gel stained with YFP-LecB (red) prior to freeze-drying. It has pores in the sub µm range^35^ which are below the resolution (Fig. 3A, top left panel). Moreover, in comparison the hydrogel is shown after freeze-drying with pores of up to 100 µm in diameter (Fig 3A, top right panel). As a fusion protein was used to trigger adhesion, it had to be proved that not the YFP part itself does initiate or influence the binding properties of the lectin domain. To do so, we used the structurally similar green fluorescent protein (GFP) as a control. Macroporous hydrogels were incubated with GFP to exclude any possible interaction with the network structures. Compared to YFP-LecB, GFP was not found on the backbone of the hydrogel but was exclusively located in the pores of the material indicating a complete lack of interaction with the matrix itself. In contrast to YFP-LecB, all GFP could be removed completely from the matrix in a single washing step, leaving no residual GFP within the gel after washing. 3D confocal images of the porous network further visualize the interconnectivity of the sponge-like material through the free diffusion of GFP, (Fig. 3B) which is important for the behavior of cells within the network: toxic metabolites are removed promptly while nutrition, growth factors and oxygen are able to reach the cell at any time^47^.

**Figure 4:**
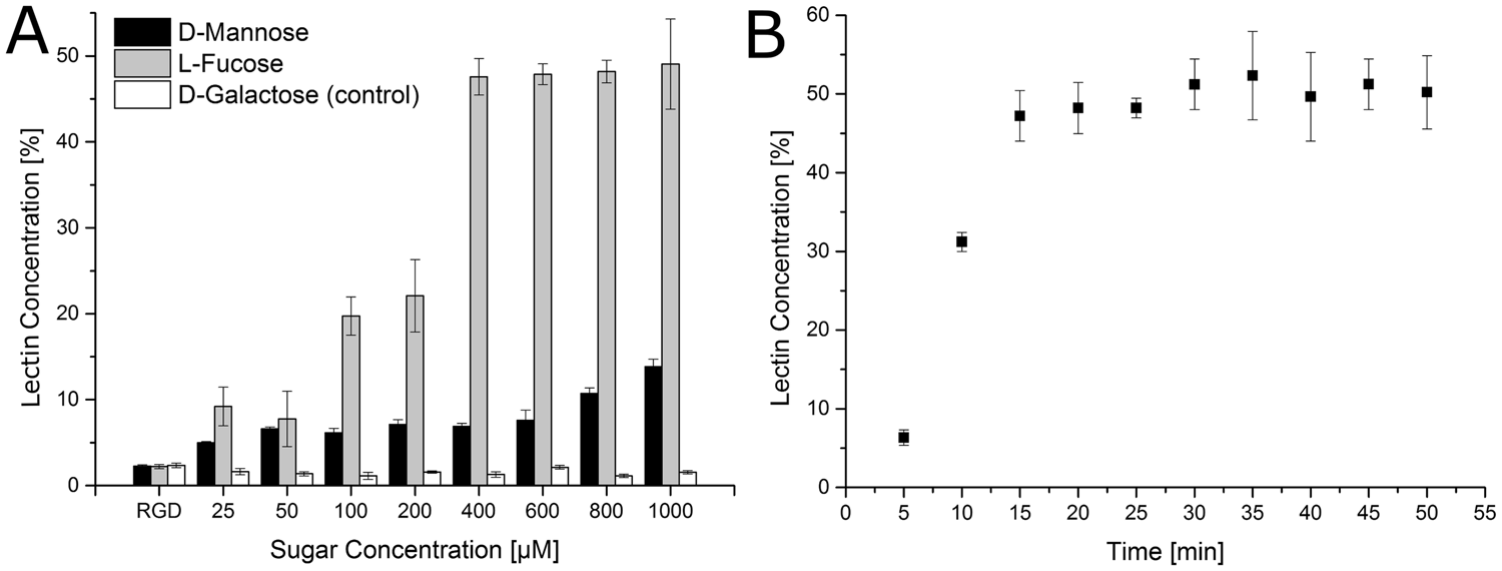
Lectin release from the hydrogel. To test the reversibility of YFP-LecB from the macroporous hydrogel matrix, gels were decorated with YFP-LecB. Afterwards, decorated hydrogels were incubated with different concentration (0–1000 µM) of lectin binding sugars (D-mannose, L-fucose and D-galactose). After 2 h, the YFP-LecB concentration was measured via fluorescence in the supernatant. (**B**) To investigate the time which is needed to elute lectin from the hydrogel, hydrogels decorated with YFP-LecB were incubated with 400 µM of L-fucose and at regular intervals samples were taken from the supernatant and analysed for YFP-LecB fluorescence. All bars represent the standard deviation.

YFP-LecB is capable of binding to the glycosylated macromolecular network hydrogel matrix by its affinity for fructose. It had to be tested if and to which extent this binding can be reverted and whether YFP-LecB can be quantitatively removed from the network by elution with sugars with a higher affinity. Thus, hydrogels were incubated with 300 µM of YFP-LecB for 10 minutes which has been shown to be sufficient for YFP-lecB binding. Afterwards, the hydrogels were incubated in a sugar-containing solution. As LecB has been shown to possess different affinities for the different sugars D-galactose, D-mannose and L-fucose^33^, these sugars were investigated for their potential to replace the bound sugar moieties and thus release LecB from the network. YFP-LecB is not able to bind the hexose D-galactose^33^, so this sugar was used as a control to prove the specificity of YFP-LecB interaction. All YFP-LecB covered gels were incubated for two hours in sugar solution of different concentrations from 0 up to 1000 µM (Fig. 4A). The relative concentration of released YFP-LecB in the supernatant was measured via fluorescence. For D-galactose, only marginal amounts of YFP-LecB were released from the material, at any of the sugar concentrations used. In contrast, D-mannose and L-fucose were functional and the protein was released with a higher efficiency for L-fucose compared to D-mannose. At the maximal sugar concentration for L-fucose, 50% of YFP-LecB were eluted compared to 15% for D-mannose (Fig. 4A). For L-fucose, about 400 µM sugar solution was enough to reach 50% lectin release from the matrix; higher concentrations did not result in higher YFP-LecB release rates. About half of the lectins bound to the network cannot be eluted, although the affinity for L-fucose is significantly higher. This effect may appear rather due to unspecific sterical hindrance in the network than to equilibrium effects. As L-fucose had the best effectivity in releasing YFP-LecB from the network, this sugar was investigated concerning the time dependency of lectin release. Every 5 minutes, a sample was taken and after only 15 minutes, a plateau with about 50% of released lectin was reached (Fig. 4B).

**Figure 5:**
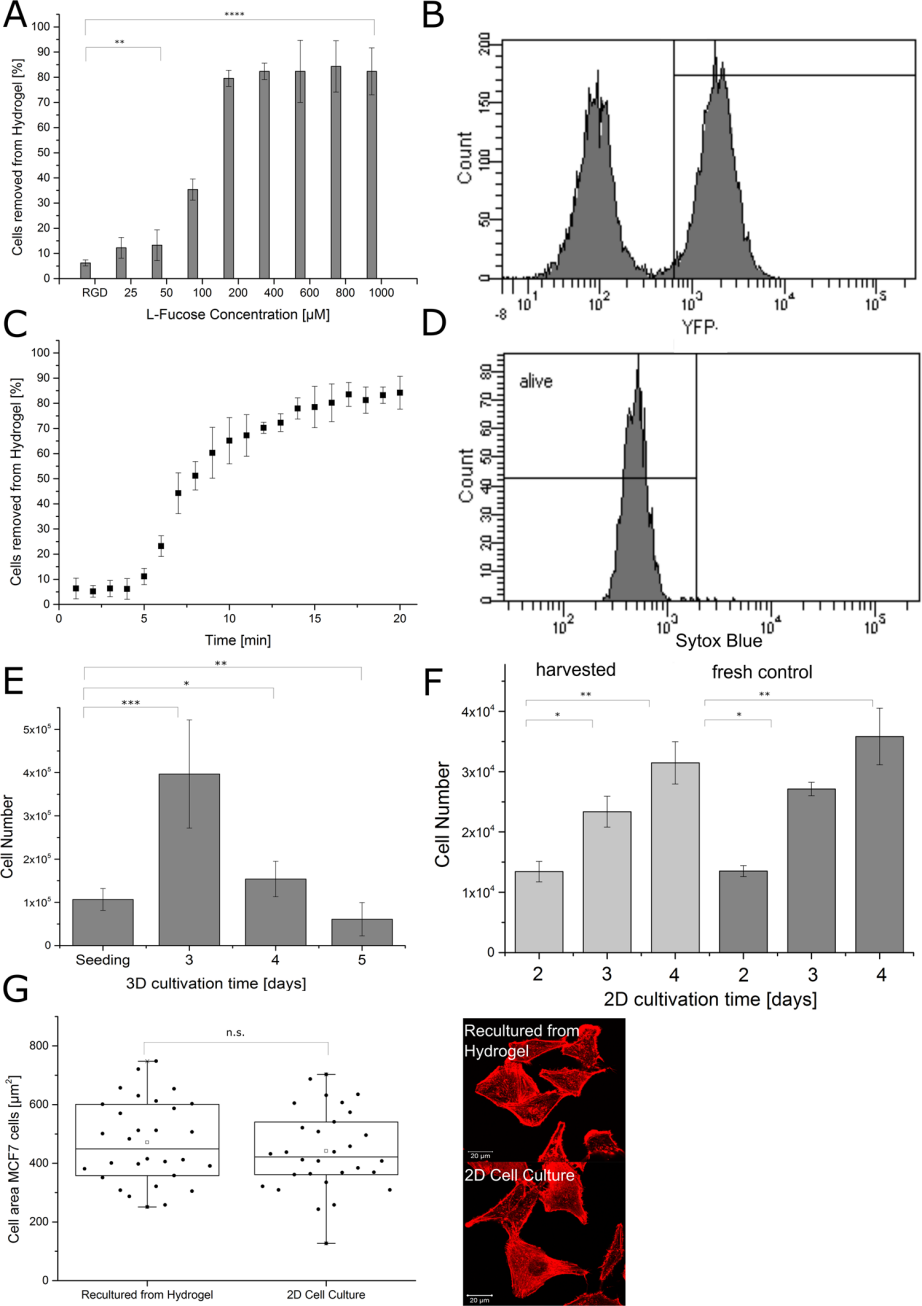
Release of A549 cells from the hydrogels. Hydrogels were polymerized and freeze-dried as described earlier and decorated with YFP-LecB. Cells were seeded into the hydrogels and incubated for 2 h to guarantee cell adhesion. (**A**) Cell loaded hydrogels were incubated with different concentrations of L-fucose and about 90% of cells could be released with 100 µM of L-fucose. (**B**) YFP-LecB after elution of the cells. Flow cytometry analysis was conducted, revealing that about 50% of all cells still bear YFP-LecB on the surface. (**C**) Cell-loaded gels were incubated with 100 µM of L-fucose, samples were taken at regular intervals and cells were counted. After approx. 10 min, 90% of the cells were eluted from the matrix. (**D**) Biocompatibility of materials and procedures used. Cells were eluted from the hydrogel and cytotoxicity was investigated with flow cytometry. (**E**) Cells were seeded and grown for 3 days in the hydrogels, eluted with 100 µM of L-fucose and counted with a Neubauer counting chamber to see proliferation of cells. (**F**) Cells were eluted from the hydrogel with 100 µM of L-fucose and re-cultured under 2D conditions over 4 days (left side) and the growth was compared with a fresh control (right side). (**G**) Adhesion of eluted cells after being re-cultured in 2D. The right side shows the average surface area of re-cultured cells compared to a fresh control. The right side shows the actin filament of re-cultured and fresh cells to observe possible differences in their morphology. All bars represent the standard deviation. The significance was tested with a one-way ANOVA with alpha = 0.05 for Fig. 5A, E and G and with a two-way ANOVA for Fig. 5F with alpha = 0.05.

Since the major goal of this study was the introduction of lectin B as an adapter molecule and useful tool to create reversible cell binding to biomaterials, we now wanted to combine the results and to demonstrate the reversibility of immobilization and behavior of cells in the glycosylated matrix. The respective hydrogels were dried at room temperature, followed by addition of a highly-concentrated cell/YFP-LecB culture medium solution onto the hydrogels. Cells penetrate the sponge-like material, diffuse through the matrix and then adhere to the backbone. After 2 h of incubation for appropriate adhesion, the cell culture medium was supplemented with L-fucose to a final concentration of 400 µM. For the release of the YFP-LecB fusion adaptor protein, previous experiments had shown 400 µM of L-fucose to be the optimal concentration to reach the maximum release and 15 minutes to be a sufficient incubation time (Fig. 4A and B). These conditions, however, do not necessarily have to be suitable for adapter mediated binding of cells as multivalency of the binding might influence immobilization and elution efficiency of cells within the network. In fact, about 90% of cells could be released by addition of L-fucose, while about 50% of the lectin still remains in the network (Fig. 4A compared to figure Fig. 5A and Supplementary Fig. S3). Interestingly, a lower L-fucose concentration was sufficient to release cells from the matrix compared to the release of the fusion protein itself. A plateau phase was already reached after the addition to a final concentration of 100 µM L-fucose, while for maximal protein release, about 400 µM of L-fucose was necessary, whereas higher L-fucose concentrations had no further effect. Moreover, cells are release much faster than the pure protein. After 10 minutes, 30% of YFP-LecB is released while about 70% of cells were already in solution supporting the hypothesis that immobilization of a single cell may require multiple YFP-LecB units. In addition, however, already incomplete replacement of individual adhesion sites was sufficient to release cells. Flow cytometry analysis revealed the distribution on YFP-LecB before and after elution from the hydrogel. Whereas all cells incubated with lectin, bear the sugar binding protein on their surfaces (Figs 1C and Supplementary Fig. S1), only 50% of the cells still bear YFP-LecB on their surface after elution from the matrix.

As cells can be integrated into the network, the capability to allow proliferation and the biocompatibility of the applied procedure and materials was further analysed. The principal feasibility of BSA in cell culture application has already vastly been investigated^48^, as well as its use as a 3D matrix for cell culture^49^. Lectins, on the other hand, in combination with hydrogels, have never been used in cell culture, but rather with analytical methods in cell culture^50–52^. To exclude the possibility that the immobilization and elution procedure might be somehow harmful to the cell, cells were eluted from the hydrogels as described, and their survival was analysed with a flow cytometry based cell viability assay. As expected, viabilities of more than 95% were obtained (Fig. 5D and Supplementary Fig. S3) after elution from the material, which shows the unrestrained biocompatibility of the used materials and procedures. The lectin offers the opportunity to bind to glycosylated protein as a backbone to immobilize cells in order to avoid negative effects on cell viability upon degradation, but these properties do not guarantee growth of cells in the immobilized state or survival of growing cells upon their release from the gel. Thus, we analysed whether cells can proliferate. Cell numbers were counted directly after seeding and after three, four and five days. Cells were seeded onto the hydrogel with an initial cell number of 10^5^ cells per well. After 3 days, cells were eluted by the described procedure from the material and the cell number was determined, showing a four-fold increase in cell numbers, indicating significant proliferation of cells in the created microenvironment. Although the amount of cells to be eluted from the material after four or five days decreased significantly, this does not necessarily mean that viability drops but is more probably caused by the formation of extracellular matrix components and structures by the cells (Fig. 5E and Supplementary Fig. S3). This is further confirmed by the fact that cells re-cultured after harvesting on day four showed the same growth behavior and rate as fresh cells from a conventional control cell culture (Fig. 5F and Supplementary Fig. S3). These cells were further analysed by seeding them on a standard cell culture flask and growing them for one day. After that, the actin cytoskeleton of cells was stained and their morphology and their surface coverage was analysed with CLSM. When compared to freshly prepared control cells from a conventional culture, the surface coverage as well as the morphology showed no significant differences, indicating that the temporary residence in the novel macroporous material at least does not alter these phenotypes.

## Discussion

3D cell culture is a key technology in modern research areas like biotechnology, biomedicine, tissue engineering, printable biotechnology and ethically superior *in vitro* diagnostics to avoid animal experimentation. In biotechnological applications for the generation of human cells as a product (e.g. leukemia treatment, stem cell applications) it is a major challenge to create suitable niches in synthetic materials and to release the cells after proliferation^4–6^. Biodegradable materials are regarded as a promising solution for many years, but the use of e.g. proteases to degrade protein based systems may be harmful to cells *per se* or may generate toxic degradation products and thus foil the intended advantages. In nature, many bacteria form biofilms with 3D architectures resembling functionalities with analogy to rudimental tissues in higher organisms by forming matrix-like scaffolds to which the bacterial cells immobilize themselves. One prominent example from the genus *Pseudomonas* is *P. aeruginosa* as the most important and best characterized species forming biofilms. *P. aeruginosa* uses the tetravalent lectin B (also known as PA-IIL)^53–55^ as natural occurring adaptor proteins to bind to extracellular matrices via their affinity for sugar residues. Sugars or their derivatives (e.g. oligosaccharides or functionalized dendrimers^56, 57^) can be used to disperse those structures and release the cells. YFP-LecB can be efficiently produced in *E. coli* ^34^ und purified via a D-mannose affinity chromatography, which makes the production easy and permits the unlimited production of the adaptor molecule. One major advantage of the affinity purification is the in-process control of the proteins functionality: the binding of YFP-LecB to the column indicates the correct folding and functionality of the tetrameric lectin B domain, while its fluorescence directly correlates with the functionality of the YFP reporter domain. The specific interaction with the hydrogel is enabled via glycosylation of the protein precursors to create multiple fucose YFP-LecB binding moieties in the structure. A very simple and almost ancient technique, the so called maillard-reaction^39^, is used to functionalize the material backbone. This simple and robust technique is not only easy to reproduce, but also offers the opportunity to determine glycosylation intensity in a time-dependent manner, while being easy to observe with a periodic acid-schiff reaction method. Those modified BSA precursors readily form hydrogels without any further change in the production procedure. Those hydrogels are not only stable over an extended period, very resistant against changing environmental conditions and rather cheap in production^35^, but further offer the possibility to create specifically tailored properties, like adjusted pore sizes and elasticity^35, 46^. Furthermore, different glycosylation intensities represent a good tool in evaluating the extent of YFP-LecB binding to the matrix (Fig. 1A). For the YFP-LecB release from the matrix, approximately four times as much YFP-LecB could be released when fucose was used compared to mannose. This can be explained with the high binding constant of 1.6 * 10^6^/M of LecB for L-fucose^33^. However, higher L-fucose concentrations did not increase the protein release from the matrix over a threshold of 50%. As all hydrogel cavities are considered to be freely accessible by protein, due to their unlimited interconnectivity in the gel and as L-fucose has a significantly higher affinity compared to the fructose on the protein backbone, it is reasonable to suspect the lectin to be released completely from the matrix. However, approximately 50% of YFP-LecB remains bound in the network, indicating an incomplete release of YFP-LecB, which may be an effect of a steric hindrance on the protein backbone surface, posing limitations to the solvent accessibility of the eluting agent. As the goal of this study was to transfer the native function of the lectin B to immobilize bacterial cells to human cells, we used one of the most widely used and well-characterized model cell lines (A549 and MCF7) in combination with the glycosylated macroporous hydrogels and the YFP-LecB to prove the principal of our concept. YFP-LecB, as the adaptor molecule, can bind to the backbone of the hydrogel and this process can be easily monitored via fluorescence of the YFP domain. As expected, cells could be immobilized in the matrix where they adhered and showed the typical increased of contact surface areas. This adhesion could be reverted and the immobilized cells were removed rapidly and efficiently upon addition of a biocompatible sugar as an elution agent. After elution of the cells from the network, about 50% of the cells still carry YFP-LecB. As cells have a large variety of sugars in their surface structures, it is possible that lectin can strongly bind to some of those sugar moieties – probably fucose – with high affinities, which is a possible explanation for the lack of elution of YFP-LecB from the cells. However, this might be a useful tool for the detection and immobilization of cells with a specific type of sugar on its surface. Furthermore, it might be possible to use this to enable a gradient elution of cells, depending on the type and affinity of sugar on its surface or to establish an assay for the detection of glycosylation pattern of cells and the subsequent separation and analysis of cells. Although there is certainly room to improve and to optimize the components used in our experiments in the non-optimized environment and procedure, already reasonably high viabilities and even significant proliferation of the cells were observed within the hydrogel matrix.

Physico-chemical properties, microarchitecture of the materials and media composition play a role in the behavior of cells within the matrix^47, 58^ and especially pore sizes and the elasticity of materials and thus their mechanical properties severely influence cell fate. The overall aim in this work was to establish and provide a new concept of reversible cell adhesion in a 3D cell culture matrix for technical cultivation and proliferation of cells. In our study cells were not only healthy and able to proliferate but could in addition be released from the material to be seeded again and re-cultured repeatedly. However, the interaction between cells and hydrogel matrix is only sugar mediated and therefore artificial because normal interactions are mediated by specific cell proteins interacting with cells or matrix components like integrins interacting with fibronectin. This sugar-mediated non-native interaction may result in altered cytoskeleton morphologies, in up- or downregulation of specific cellular metabolic pathways or deviating cell-cell interaction. This artificial nature of interactions represents a general limitation of the new system for systematic biological studies of native cell behavior and physiology while this technique may be useful in biotechnological applications for multiplication of cells and their harvest. Proliferation and successful release was possible for limited periods of a few days. Elongated periods, however, led to drastically reduced efficiencies of cell release, which are probably the result of extensive growth and the resulting dense packing of cells within the cavities avoiding or at least reducing their release from the matrix. Although the A549 and MCF7 cancer cells used were model cell lines characterized by their robustness, the experiments presented here represent a proof of concept which will be extended to more sensible cells like hematopoetic stem cells or other cells typically requiring specific niches for proper cultivation. Our technique is inspired by the events taking place in bacterial biofilm formation and can be used to guarantee initial attachment of cells to a matrix which can provide any 3D architecture and elasticity required for the creation of artificial niches. Although the lectin mediated interactions do not resemble naturally occurring cell-matrix interactions (e. g. via integrins) the cells showed inconspicuous morphologies indicating sound conditions in the environment of our material. Whenever cells are used as a product, the biocompatibility of all chemicals, materials and procedures used in the process may play a tremendous role and influence the outcome and value, especially if products and procedures aim towards application in the field of biomedical science. Endotoxin levels, for example, are among the key criteria of a save product in proper condition. The YFP-LecB fusion protein did not show relevant toxicity and the endotoxin levels were as low as ~1.1 EU/ml, which has been shown to be harmless for cells^45^ (Fig 1C). As our study shows, the tetrameric structure of YFP-LecB can be used as an adaptor molecule to realize the binding of human cells to an extracellular matrix via its affinity for sugars. This procedure offers new approaches for the creation of sophisticated new materials with reversible cell adhesion properties. Although the concept to use a lectin for reversible cell immobilization has been demonstrated with YFP-LecB in our study, it is very clear that in principle every other lectin with more than one binding site can replace our adaptor molecule. There are probably several hundreds of lectins available or will become available in this context, each potentially offering unique and valuable properties and a wide range of different affinities for a variety of sugars. One prominent example, used for decades in biomolecular and cell sciences, is concanavalin A^59^ which will certainly be an alternative.

The major aim of this study was to transfer the concept for reversible lectin-based cell immobilization in bacterial biofilms into a technique to immobilize cells within a matrix, by using a combination of a biodegradable and highly biocompatible protein based hydrogel and a system of very simple glycosylation, providing accessible fructose and a YFP-LecB containing synthetic adaptor protein for specific binding. Although the lectin-sugar interaction must be considered as artificial, with this bioinspired approach we have implemented a novel technique to reversibly immobilize cells within this macroporous system, which may develop into a more general technique for the development of methods and bioreactors for the cultivation and harvesting of cells.

## Material and Methods

BSA was purchased from Sigma-Aldrich (St. Louis, Missouri, USA) and appropriate stocks were created prior to use. THPC was purchased from Sigma-Aldrich (St. Louis, Missouri, USA), dilutions were stored at room temperature (RT). Trypsin-Ethylenediaminetetraacetic acid (EDTA) (0.05% (w/v)), 2-propanol, D-mannose, L-fucose, D-galactose were purchased from Sigma-Aldrich (St. Louis, Missouri, USA), Trypan blue, Dulbecco modified eagle medium (DMEM), Phosphate Buffered Saline (PBS), Penicillin-Streptomycin (1000 U/mL), and non-essential amino acid solution (MEM) were purchased from life technologies (Carlsbad, California, USA), Hoechst fluorescent dye (H1399), Rhodamine B, SytoxBlue and limulus amoebocyte lysate (LAL) detection kit were purchased from Thermo Fisher Scientific (Waltham, Massachusetts, USA) and formaldehyde from Carl Roth (Carl Roth GmbH und Co. KG, Karlsruhe, Germany).

### Lectin production and purification

For each experiment the expression strain was freshly transformed with 1 µl YFP-LecB plasmid^34^ (180 ng/µl) being mixed with chemical competent *E. coli BL21 Tuner* (Merck Millipore, Darmstadt, Germany), incubated 45 min on ice followed by 42 °C for 1 min and subsequent incubation on ice for 3 min 800 µl of pre-warmed lysogeny broth (LB) medium was added and cells were incubated at 37 °C for 1 h, followed by plating 50 µl of cell suspension on selective LB-amp (100 µg/ml) medium and incubation over night at 37 °C. The next day, one clone was picked and transferred to 50 ml selective LB-amp medium and grown at 37 °C overnight. The next day, expression cultures were inoculated with an optical density (OD) of 0.1 in 200 ml (0.4% glucose and 100 μg/mL ampicillin) in a 2 L flask and grown at 37 °C to an OD of 0.6 followed by induction with 1 mM isopropyl-β-d-thiogalactoside (IPTG) and a temperature shift to RT. After 16 h of cell growth, cells were harvested by centrifugation at 3.000 × g, 4 °C for 30 min and suspended in 15 ml of 100 mM Tris-HCL (1 mg/ml Lysozyme) pH 8.0, incubated for 30 minutes on ice, sonicated for 15 min at 40% intensity (6 cycles) on ice, followed by centrifugation at 10.000 × g, 4 °C for 30 min. Purification of YFP-LecB by affinity chromatography (D-mannose Agarose Beads M6400 Sigma Aldrich, St. Louis, Missouri, USA) was carried out at 37 °C; the column was equilibrated with 3 ml of 100 mM Tris-HCl, pH 8.0, and the cell extract was loaded onto the column and incubated for 1 h at 37 °C, followed by subsequent washing with 15 mL 100 mM Tris-HCl, 150 mM NaCL, pH 8.0. The YFP-LecB fusion protein was eluted with 4 bead volumes of 100 mM Tris-HCl, pH 8.0, containing 20 mM D-mannose. Samples were further concentrated using Vivaspin 20 microconcentrators (Mr cutoff, 10 kDa) and washed with 100 mM Tris-HCl, pH 8.0. The amount of protein was determined with the specific absorption coefficient at 280 nm and monitored via fluorescence measurements at 514 nm.

### Glycosylation of BSA

Glycation of BSA was conducted following a modified protocol from Ledesma-Osuna *et al*.^38^. In brief, 5 ml of BSA (20 mg/ml) was mixed with a 5 ml saccharose solution (40 mg/ml), followed by addition of 5 ml 0.1 M phosphate buffer, pH 8.0. Samples were frozen in liquid nitrogen and freeze-dried (FreezeDryer Epsilon 1–6D, Christ, Osterode am Harz, Germany) over night. Samples were then heated to 60 °C for 30, 60 and 120 min respectively. Glycosylation was determined using a periodic acid-schiff reagent method, following the manufacturer’s instructions (Pierce™ Glycoprotein Staining Kit, ThermoFisher Scientific, Waltham, USA), resulting in magenta colored bands of the glycosylated protein.

### Preparation of glycosylated hydrogels

Hydrogels were prepared as we described earlier^35, 46^. Briefly, THPC solution was mixed with the same volume of glycosylated BSA (20% (w/v)) in PBS, resulting in polymerization within minutes by formation of covalent bonds between primary amines and hydroxy groups, followed by subsequent freeze-drying overnight (FreezeDryer Epsilon 1-6D, Christ, Osterode am Harz, Germany) to generate pores of a 10–100 µm within the gel structures by sublimation of ice crystals from the network, leaving empty, air-filled spaces within the network.

### Lectin binding to cells and hydrogels

To investigate the binding of LecB to A549 and MCF7, 2*10^4^ cells were seeded onto an 8 well µ ibidi slide (ibidi GmbH, Munich, Germany) and grown over night. After 24 h of cell growth and adhesion, cells were fixed with 3.7% (w/v) formaldehyde, washed twice with PBS, stained with 5 μl of a 300 unites of rhodamine-phalloidin solution in 195 μl PBS for 30 min, washed twice with PBS and stained for 10 min with 1 µl DAPI in 199 µl PBS. Afterwards, cells were incubated for 10 minutes with 200 µM YFP-LecB and washed twice with PBS to remove unspecific bound molecules. Areas of the cell cross sections were investigated with a Zeiss Confocal Microscope (Carl Zeiss Ag, Oberkochen, Germany) at 561 nm for cytoskeleton (phalloidin-rhodamine), 400 nm for cell nucleus (DAPI) and 514 nm for YFP-LecB. To visualize YFP-LecB binding to hydrogels, glycosylated and non-treated hydrogels were stained with YFP-LecB for 10 minutes, washed twice with PBS and observed at the Zeiss Confocal Microscope (Carl Zeiss Ag, Oberkochen, Germany) at 488 nm. To investigate the extent of LecB binding to the cells, cells were incubated with 300 µM YFP-LecB for 30 min, followed by analysis with a BD LSR Fortessa^TM^ (BD Pvt. Limited, Gurgaon, Haryana, India) with FACS software (BD FACSDiva™) using a standard protocol. To investigate the biocompatibility of LecB, cells were incubated in cell culture medium for 24 h with 300 µM of LecB, followed by analysis with a BD LSR Fortessa™.

### Visualization of porous hydrogels and specificity of lectin B binding

In order to visualize porous hydrogel structures, untreated (not freeze-dried after polymerization) and freeze-dried hydrogels were incubated with 200 µM YFP-LecB, washed twice with PBS, cut into small fragments and analysed at the Zeiss Confocal Microscope (Carl Zeiss Ag, Oberkochen, Germany) at 488 nm (Fig. 3). To investigate YFP-LecB specificity for the hydrogels, the YFP-LecB gels were diffused by 200 µM GFP for 1 min and analysed at the Zeiss Confocal Microscope (Carl Zeiss AG, Oberkochen, Germany) at 488 and 514 nm.

### Seeding of cells onto hydrogels

Hydrogels were polymerized as described earlier, freeze-dried and washed for 1 day in PBS to remove all unreacted components, followed by subsequent drying of hydrogels at RT. Afterwards, 2*10^5^ cells were incubated with YPF-LecB for 30 min in PBS. In the next step, cells were seeded onto dried gels, resulting in absorption of cell loaded solution into the porous hydrogel matrix. After 15 minutes of incubations, cell loaded gels are transferred into a 12 well plate and covered with 2 ml DMEM (FBS, 10% (w/v), 1% (w/v) Pen-Strep 1% MEM (w/v)) and incubated at 37 °C, 5% CO_2_ moisturized atmosphere for appropriate times, depending on the assay.

### Sugar specificity and release of lectin B from hydrogels

Porous hydrogels were prepared as described earlier and incubated with a 300 µM YFP-LecB solution for 30 min, followed by transferring LecB loaded gels into 2 ml PBS solution, containing 0; 25; 50; 100; 200; 400; 600; 800 and 1000 µM of D-mannose, L-fucose and D-galactose respectively. After 2 h, hydrogels were removed from solution and solution was observed for YFP-LecB concentration with a Tecan200M fluorescence reader (Tecan Group Ltd., Männedorf, Switzerland) at excitation wavelength of 509 nm and emission wavelength of 532 nm respectively for all sugar concentrations. As L-fucose resulted in the best YFP-LecB release, L-fucose was investigated for time-dependency. Hydrogels were incubated in 300 µM LecB solution, transferred into 2 ml of PBS, containing 400 µM L-fucose. Every 5 minutes, samples were taken and YPF-LecB concentration was determined via fluorescence measurements.

### Harvesting of cells by sugar mediated elution from hydrogels

Cell release was investigated with the concentrations of 300 µM of YFP-LecB and 400 µM of L-fucose determined prior. Hydrogels were prepared and loaded with cells as described earlier and incubated in DMEM (FBS, 10% (w/v), 1% (w/v) Pen-Strep 1% MEM (w/v)) at 37 °C, 5% CO_2_ moisturized atmosphere for 2 h. Samples were analysed for unspecific cell release, followed by addition of L-fucose to a final concentration of 400 µM. In the release assay, cells were incubated for another 2 h followed by 5 min of shaking (30 rpm) and removal of hydrogel. Solutions were centrifuged for 3 min at 700 rpm and followed by cell counting with a Neubauer counting chamber (Celeromics, Milton Road, Cambridge, U.K.). In the second, time-dependent assay, cell-loaded hydrogels were incubated in the presence of 400 µM L-fucose for different times, before harvesting and counting the cells as described in this paragraph.

### Cell proliferation and flow cytometry cytotoxicity assay

To find out about cell proliferation, hydrogels were seeded with cells as described earlier and incubated in DMEM (FBS, 10% (w/v), 1% (w/v) Pen-Strep 1% MEM (w/v)) at 37 °C, 5% CO_2_ moisturized atmosphere for appropriate times (up to 5 days) and harvested with 400 µM L-fucose. Cell containing solutions were centrifuged at 700 rpm, 3 min and counted with Neubauer counting chamber (Celeromics, Milton Road, Cambridge, U.K.) to reveal proliferation and elution rates. To investigate about biocompatibility and the whereabouts of YFP-LecB, in a first approach cells are incubated with 300 µM YFP-LecB for 30 min followed by subsequent staining with 0,05 µg/ml of SytoxBlue (Thermo Fisher Scientific, Waltham, Massachusetts, USA) for 5 min, 10^5^ cells were analysed with a BD LSR Fortessa^TM^ (BD Bioscience, Becton, 1 Becton Drive, New Jersey, USA).

### Re-culturing of eluted cells

1*10^5^ cells were seeded into the hydrogel as described earlier. After 4 day in the hydrogel, cells were eluted with 400 µM of L-fucose and 5*10^4^ cells were re-cultured in a 25 cm^2^ adherent plate. Fresh cells were seeded in another 25 cm^2^ plate. After 2, 3 or 4 days, cells were harvested with 0.05% trypsin-EDTA and counted with a Neubauer counting chamber. The growth of eluted and fresh cells was compared. To investigate the morphology of the cells, cells were eluted and re-cultured in 2D. After 24 h of cell growth and adhesion, cells were fixed with 3.7% (w/v) formaldehyde, washed twice with PBS, stained with 5 μl of a 300 unites of rhodamine-phalloidin solution in 195 μl PBS for 30 min and areas of the cell cross sections were investigated with a Zeiss Confocal Microscope (Carl Zeiss Ag, Oberkochen, Germany) at 561 nm to visualize the cytoskeleton. The surface of the cells was measured, plotted and compared to fresh cells.

### Endotoxin testing

The endotoxin levels of purified protein fractions were detected with a LAL chromogenic endotoxin quantitation kit (88282) from Thermo fisher scientific (Waltham, Massachusetts, USA). 50 µl of sample was used, following manufacturers instruction and absorption was detected with a Tecan200M fluorescence reader (Tecan Group Ltd., Männedorf, Switzerland) at 407 nm.

### Statistical analysis

All bars in the graphs represent standard deviation from triplicates. For variance analysis, either one-way or two-way ANOVA was applied.

## Electronic supplementary material

Supplementary Information
