# Supplementary material for: Lectin-mediated reversible immobilization of human cells into a glycosylated macroporous protein hydrogel as a cell culture matrix
Source: Sci Rep. 2017 Jul 21;7:6151. doi: 10.1038/s41598-017-06240-w (PMC5522389; doi:10.1038/s41598-017-06240-w)
Supplement: Supplementary file 1 — Supplementary Information [file 41598_2017_6240_MOESM1_ESM.pdf]

Lectin-mediated reversible immobilization of human cells into a glycosylated macroporous protein hydrogel as a cell culture matrix

Nicholas Bodenberger[1], Dennis Kubiczek[1], Laura Trösch[1], Ali Gawanbacht[2], Susanne Wilhelm[3], Denis Tielker[4], Frank Rosenau[1]*

[1] Center for Peptide Pharmaceuticals, Faculty of Natural Science, 89081 Ulm University, Germany

[2] Core Facility Flow Cytometry, 89081 Ulm University, Germany

[3] Heinrich Heine Universität Düsseldorf, Deans Office, 40204 Düsseldorf, Germany

[4] QIAGEN GmbH - Germany, Qiagen Straße 1, 40724 Hilden, Germany

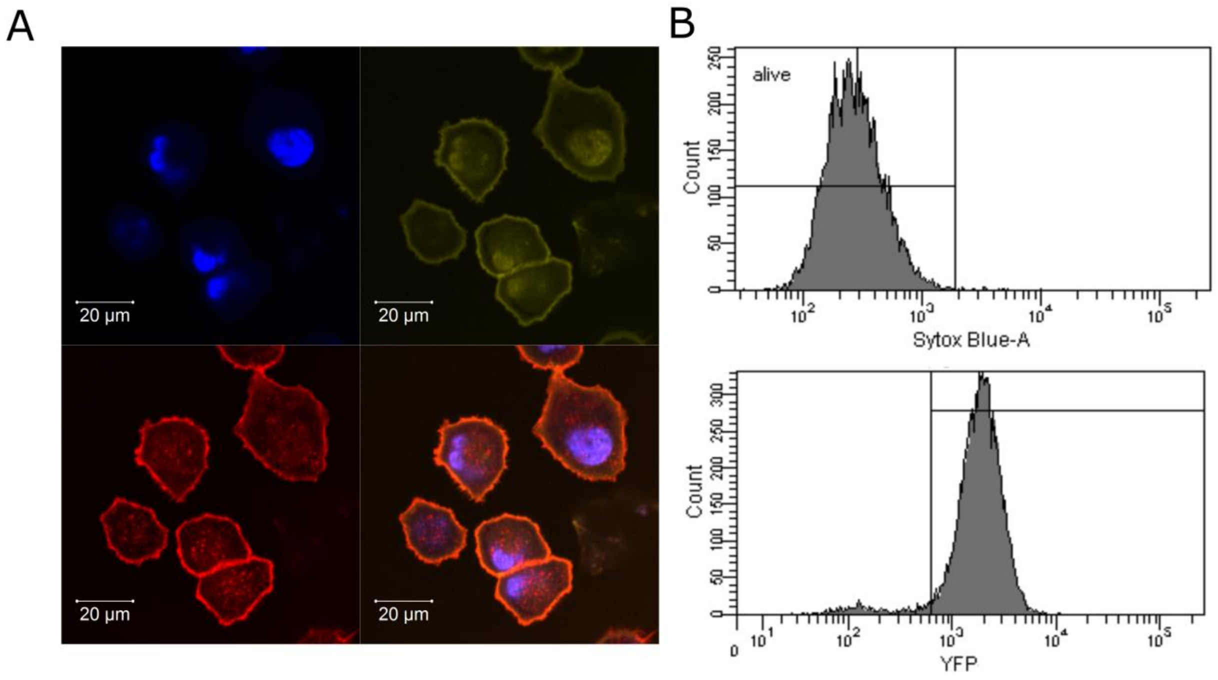

S1 **Decoration of cells and hydrogels with YFP-LecB and biocompatibility**. (A) Breast cancer cells (MCF7) were incubated with YFP-LecB, washed and visualized with confocal microscopy to reveal lectin binding to the cells whereas red color represents the rhodamine-phalloidin stained cytoskeleton, blue the DAPI stained cell nucleus and yellow the YFP-LecB. (B) Cells were incubated with YFP-LecB and Sytox Blue to reveal the extent of YFP-LecB binding to the cells and its biocompatibility; YFP-LecB was proved to be biocompatible (overall viability >95% after 24h) and to bind to nearly all cells.

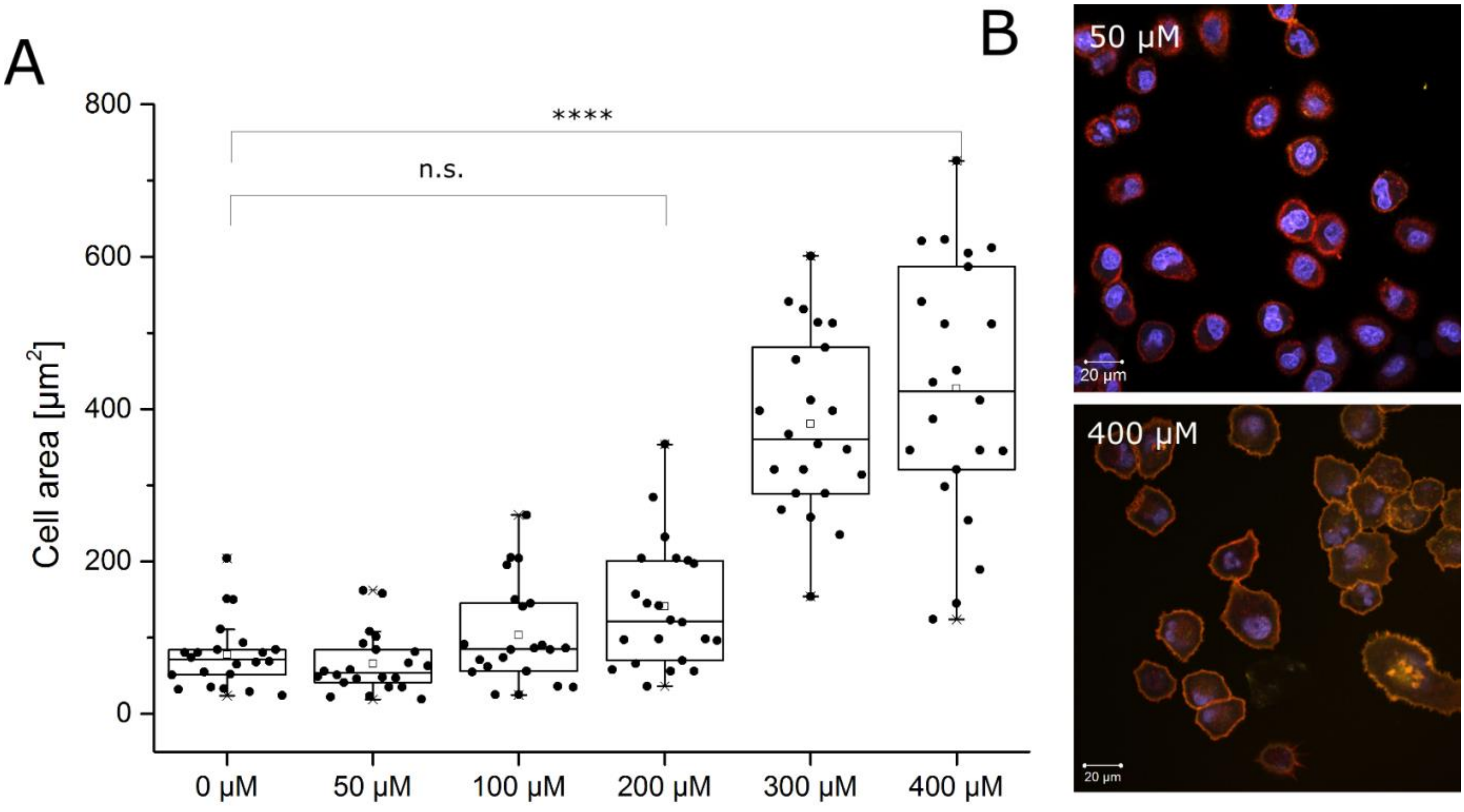

S2 **Lectin-mediated cell adhesion**. (A) 2*10^5 MCF7 cells were seeded onto a hydrogel surface in the absence or presence of different YFP-LecB concentrations from 0 to 400 µM. Cells could adhere to the surface in the presence of cell culture medium for 24 h, followed by staining of cell cytoskeleton (red phalloidin-rhodamine) and nucleus (blue DAPI). Cellular surfaces were analyzed with confocal laser scanning microscopy. Adhesion was detected for YFP-LecB concentration of 300 µM and higher. (B) Typical MCF7 cells in the presence of 50 and 400 µM of YFP-LecB. All bars represent standard deviation. The significance was determined with a one-way anova with alpha = 0.05.

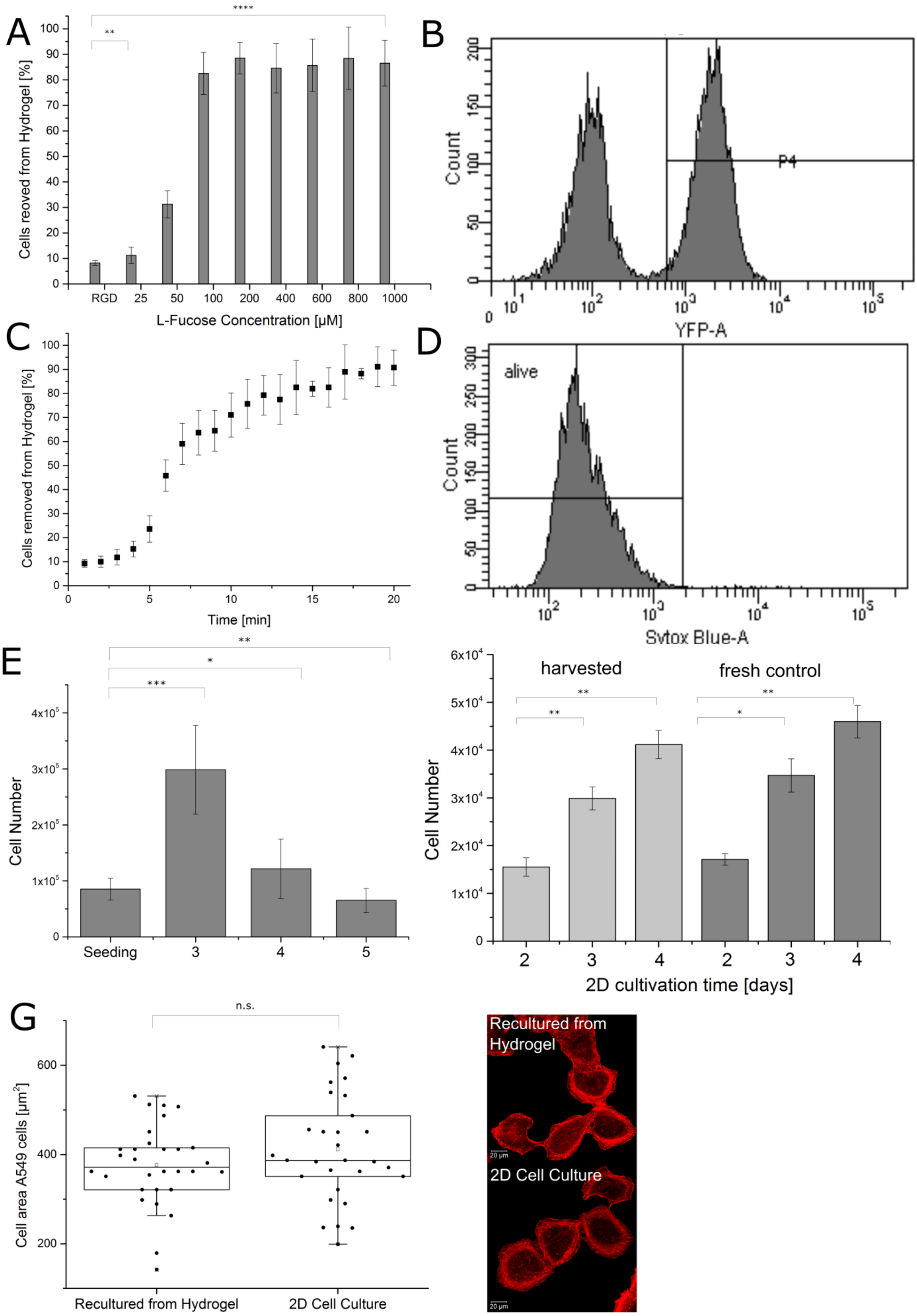

S3 **Release of MCF7 cells from the hydrogels**. Hydrogels were polymerized and freeze-dried as described in the text and decorated with YFP-LecB. Cells were seeded into the hydrogels and incubated for 2 h to guarantee cell adhesion. (A) Hydrogels loaded with MCF7 cells were incubated with different concentrations of L-fucose and about 90% of cells could be released with 100 µM of L-fucose. (B) YFP-LecB after elution of the cells.

Flow cytometry analysis was conducted, revealing that about 50% of all cells still bear YFP-LecB on the surface. (C) Cell-loaded gels were incubated with 100 µM of L-fucose, samples were taken at regular intervals and cells were counted. After approx. 10 min, 90% of the cells were eluted from the matrix. (D) Biocompatibility of materials and procedures used. Cells were eluted from the hydrogel and cytotoxicity was investigated with flow cytometry. (E) Cells were seeded and grown for 3 days in the hydrogels, eluted with 100 µM of L-fucose and counted with a Neubauer counting chamber to see proliferation of cells. (F) Cells were eluted from the hydrogel with 100 µM of L-fucose and re-cultured under 2D conditions over 4 days (left side) and the growth was compared with a fresh control (right side). (G) Adhesion of eluted cells after being re-cultured in 2D. The right side shows the average surface area of re-cultured cells compared to a fresh control. The right side shows the actin filament of re-cultured and fresh cells to observe possible differences in their morphology. All bars represent standard deviation. The significance for Fig. S3 A, E and G was tested with a one-way ANOVA with alpha = 0.05 and Fig. S3 F with a two-way ANOVA with alpha = 0.05.

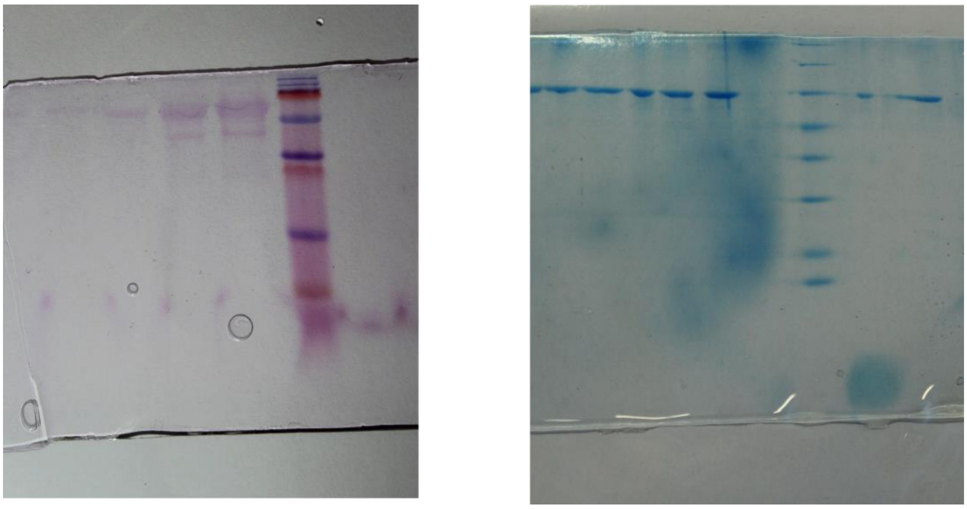

S4 SDS-pages of Figure 1A in full length. Glycosylation of BSA was determined with an acid-schiff reaction. BSA was analysed on a SDS page (right) and a glycoprotein detection gel (left) for different glycosylation times (30, 60 and 120 min) where glycosylation is visualized by magenta bands in the gel.
